# Al_2_O_3_ Layers Grown by Atomic Layer Deposition as Gate Insulator in 3C-SiC MOS Devices

**DOI:** 10.3390/ma16165638

**Published:** 2023-08-15

**Authors:** Emanuela Schilirò, Patrick Fiorenza, Raffaella Lo Nigro, Bruno Galizia, Giuseppe Greco, Salvatore Di Franco, Corrado Bongiorno, Francesco La Via, Filippo Giannazzo, Fabrizio Roccaforte

**Affiliations:** Consiglio Nazionale delle Ricerche, Istituto per la Microelettronica e Microsistemi (CNR-IMM), Strada VIII 5, Zona Industriale, 95121 Catania, Italy; patrick.fiorenza@imm.cnr.it (P.F.); raffaella.lonigro@imm.cnr.it (R.L.N.); bruno.galizia@imm.cnr.it (B.G.); giuseppe.greco@imm.cnr.it (G.G.); salvatore.difranco@imm.cnr.it (S.D.F.); corrado.bongiorno@imm.cnr.it (C.B.); francesco.lavia@imm.cnr.it (F.L.V.); filippo.giannazzo@imm.cnr.it (F.G.); fabrizio.roccaforte@imm.cnr.it (F.R.)

**Keywords:** high-κ, dielectrics, ALD, WBG, 3C-SiC

## Abstract

Metal-oxide-semiconductor (MOS) capacitors with Al_2_O_3_ as a gate insulator are fabricated on cubic silicon carbide (3C-SiC). Al_2_O_3_ is deposited both by thermal and plasma-enhanced Atomic Layer Deposition (ALD) on a thermally grown 5 nm SiO_2_ interlayer to improve the ALD nucleation and guarantee a better band offset with the SiC. The deposited Al_2_O_3_/SiO_2_ stacks show lower negative shifts of the flat band voltage V_FB_ (in the range of about −3 V) compared with the conventional single SiO_2_ layer (in the range of −9 V). This lower negative shift is due to the combined effect of the Al_2_O_3_ higher permittivity (ε = 8) and to the reduced amount of carbon defects generated during the short thermal oxidation process for the thin SiO_2_. Moreover, the comparison between thermal and plasma-enhanced ALD suggests that this latter approach produces Al_2_O_3_ layers possessing better insulating behavior in terms of distribution of the leakage current breakdown. In fact, despite both possessing a breakdown voltage of 26 V, the T-ALD Al_2_O_3_ sample is characterised by a higher current density starting from 15 V. This can be attributable to the slightly inferior quality (in terms of density and defects) of Al_2_O_3_ obtained by the thermal approach and, which also explains its non-uniform dC/dV distribution arising by SCM maps.

## 1. Introduction

The cubic polytype of silicon carbide (3C-SiC) has a smaller energy gap (E_g_ = 2.36 eV) [1,2] compared to the hexagonal 4H-SiC (E_g_ = 3.26 eV) [3], but it possesses a higher electron mobility and saturation velocity [4,5,6,7,8]. Moreover, it exhibits a larger conduction band offset (3.7 eV) [9] with SiO_2_ than 4H-SiC (2.7 eV). Hence, differently from the 4H-SiC/SiO_2_ system where they are aligned with the conduction band edge of 4H-SiC, the near-interface-oxide-traps (NIOTs) inside the insulator in the 3C-SiC/SiO_2_ system lie above the Fermi level and hence they are electrically inactive [10,11]. Furthermore, the lower position of the 3C-SiC conduction band edge with respect to the SiO_2_ conduction band edge results immune to the interface states that are peculiar of the SiO_2_/4H-SiC interface [6]. This can lead to a higher inversion electron channel mobility (>200 cm^2^ V^−1^ s^−1^ [12]) in 3C-SiC Metal-Oxide-Semiconductor Field-Effect Transistors (MOSFETs) compared to those fabricated using the 4H-poly-type.

Silicon dioxide (SiO_2_) is the native oxide of SiC that can be obtained by a thermal oxidation process of the material [13,14]. However, its electrical behavior is adversely affected by the large number of defects [9,15] (e.g., carbon clusters and dangling bonds produced during oxidation), which results in a large negative shift of the flat band voltage (V_FB_) [8,16,17]. Another issue is the response of the MOS system to the application of high voltages. In particular, in blocking configuration, the distribution of the electric field inside the insulator (E_ins_) and the semiconductor (E_s_) can be expressed by the Gauss’ law, E_ins_ = (κ_s_/κ_ins_)E_s_, where κ_ins_ and κ_s_ are the insulator and semiconductor permittivity values. Considering the permittivity values of SiO_2_ (3.9) and 3C-SiC (9.7), the SiO_2_ layer is subjected to an electric field 2.5 higher than 3C-SiC. Hence, the SiO_2_ gate insulator reliability is seriously compromised under high electric field. Moreover, using SiO_2_ does not enable full exploitation of the high critical field of the underlying 3C-SiC substrate. Consequently, thicker drift layer must be used, which in turn increases the total device on-resistance [18].

Insulators with high permittivity (the so-called “*high-κ*”) can be a solution to overcome this limitation due to the better distribution of the electric field in the MOS system, which offers safer operating conditions in high voltage applications. Al_2_O_3_ is a suitable *high-κ* oxide due to its permittivity value (κ ~ 9), good thermal stability and relatively large band gap (~7 eV) [19,20,21,22,23]. The Atomic Layer Deposition (ALD) [24,25] is the best technique for the deposition of Al_2_O_3_ thin layers with optimal thickness control, uniformity on large area, and high-quality interface [26,27,28]. The ALD growth of Al_2_O_3_ thin films on SiC can be improved by the insertion of a nanometric SiO_2_ interlayer (IL), which provides a larger amount of active nucleation sites than the bare SiC surface. Moreover, the introduction of SiO_2_ IL between Al_2_O_3_ and SiC is also convenient to guarantee a larger conduction band offset and finally to better prevent leakage phenomena [29,30]. To date, the Al_2_O_3_ deposited by ALD as gate dielectric on 3C-SiC is completely unexplored. Actually, it has been adopted by R. Oka et al. [16] only as a thin interlayer between SiO_2_ and 3C-SiC to improve the structural quality of their interface.

In this work, we report on the Al_2_O_3_ thin film growth by ALD as an alternative insulator layer for 3C-SiC MOS capacitors using a very thin SiO_2_ film as IL. In particular, the structural properties of Al_2_O_3_/SiO_2_ stacks, of their interfaces on the underlying 3C-SiC but also their electrical behavior have been investigated by comparing the two different ALD approaches, namely the thermal (T-) and plasma-enhanced (PE-) ALD processes [31,32]. Both approaches allow obtaining good quality high-κ dielectrics. However, some literature works [16,22,23,31,33,34] report on slight differences both in the quality of the grown high-κ and in its interfacial properties, directly related to the different oxidation mechanism between the two methods. In particular, the studies conducted on other semiconductor materials [16,32] suggest that the more reactive action of the O_2_-plasma produces Al_2_O_3_ layers characterised by a higher mass density and lower amounts of the undesired carbon contaminations and unreacted OH- groups, which could act as active centres for electron trapping [35].

Furthermore, the combination of several characterisation techniques, i.e., morphological-structural and electrical—either at a macroscopic scale or at a nano-scale—allowed the full comprehension of the insulating properties of the differently ALD deposited Al_2_O_3_ films.

## 2. Materials and Methods

A 10.2 µm thick 3C-SiC grown by chemical vapour deposition on Si (100) was employed as substrate [36]. Prior to the oxidation process, the 3C-SiC substrates were cleaned for ten minutes in an H_2_SO_4_:H_2_O_2_ = 3:1 solution followed by ten minutes of etching in an HF:H_2_O = 1:5 solution. A 5 nm SiO_2_ IL was grown by a controlled dry oxidation process at 1150 °C for 5 min. Successively, the Al_2_O_3_ layers were deposited on SiO_2_/3C-SiC by either thermal- or plasma-enhanced ALD using trimethylaluminum (TMA) as an aluminium precursor and H_2_O or O_2_-plasma as co-reactants. Both processes were carried out at the deposition temperature of 250 °C. Meanwhile, the different growth rates of the T-ALD (~0.9 Å/cycle) and the PE-ALD (~1.2 Å/cycle) involve the use of a different number of deposition cycles (350 and 250 cycles for T- and PE-, respectively) to grow an A_2_O_3_ layer with the same thickness of 30 nm.

The structural quality of Al_2_O_3_/SiO_2_/3C-SiC stacks and their morphology were investigated by transmission electron microscopy (TEM) using a FEG-TEM JEOL 2010F (Tokyo, Japan) microscope and by atomic force microscopy (AFM) using a DI3100 equipment by Bruker (Billerica, MA, USA) with Nanoscope V controller, respectively. In particular, the TEM analysis was carried out in cross-section in order to visualize the properties of the Al_2_O_3_/SiO_2_ stack layer and their interfaces. For this purpose, cross-sectional specimens were properly prepared both for T- and PE-Al_2_O_3_/SiO_2_/3C-SiC samples by conventional mechanical preparation techniques, i.e., including polishing and dimple grinding, followed by a final thinning with ion milling.

The electrical behaviour of the insulating stacks was evaluated by capacitance-voltage (C-V) and current-voltage (I-V) measurements carried out on lateral metal-oxide-semiconductor (MOS) capacitors using a Microtech Cascade probe station equipped with a Keysight B1505 parameter analyser (Santa Rosa, CA, USA). Finally, the nanoscale electrical behaviour of the systems was monitored my means of scanning capacitance microscopy (SCM).

## 3. Discussion

**Figure 1:**
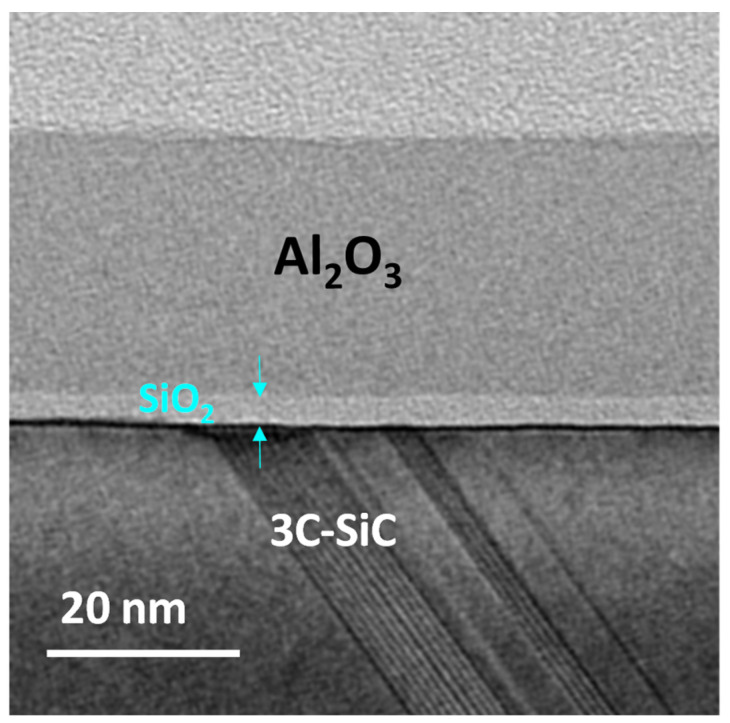
Cross-section TEM image relative to T-Al_2_O_3_/SiO_2_/3C-SiC.

The cross-section TEM image reported in Figure 1 illustrates a uniform and amorphous Al_2_O_3_ layer with a thickness of ~30 nm and a sharp interface with the underlying SiO_2_/3C-SiC. The SiO_2_-IL is clearly distinguishable and has a thickness of about 4.5 nm. The structural properties of Al_2_O_3_ and of its interfaces are similar on both T-Al_2_O_3_/SiO_2_/3C-SiC and PE-Al_2_O_3_/SiO_2_/3C-SiC systems; thus, only the first is reported representatively.

**Figure 2:**
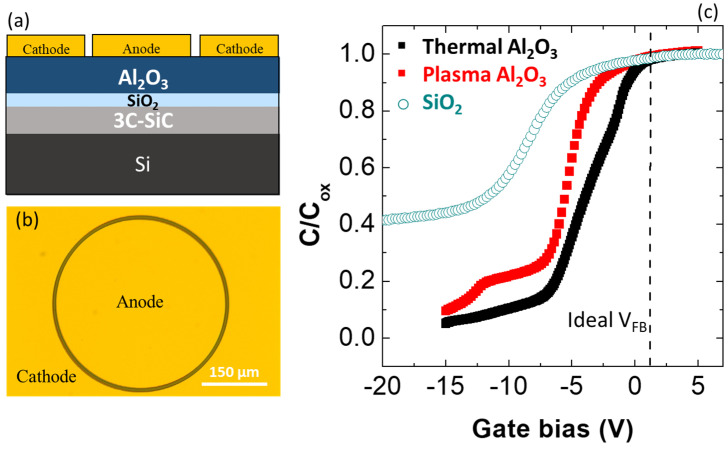
Schematic cross-section of the Al_2_O_3_/SiO_2_/3C-SiC MOS capacitor (**a**) and top-view microscopy image of a MOS capacitor (**b**). C-V curves of T-Al_2_O_3_/SiO_2_(IL)/3C-SiC and PE-Al_2_O_3_/SiO_2_(IL)/3C-SiC MOS capacitors in comparison with the analogous SiO_2_/3C-SiC (**c**).

Lateral MOS capacitors schematically depicted in Figure 2a were fabricated using photolithography, metal deposition and lift-off processes. The anode of the MOS capacitors was surrounded by a large-area metal cathode so that its capacitance could be neglected (Figure 2b). Ni/Au was used as metal electrode. The MOS structures fabricated on T-Al_2_O_3_/SiO_2_/3C-SiC and PE-Al_2_O_3_/SiO_2_/3C-SiC were probed by C-V measurements, which are shown in Figure 2c. Both samples provide C-V curves negatively shifted compared to the ideal value V_FB_ = +0.9 V. In particular, the experimental flat band voltage values were −0.6 V and −3 V for the T-ALD and PE-ALD stacks, respectively. However, as can be observed in Figure 2c, such negative shifts were smaller compared to that of MOS capacitor where the insulator was only a thick (40 nm) thermal SiO_2_ [37]. This experimental finding is related to the higher dielectric constant of the Al_2_O_3_ (κ = 8) with respect to that of SiO_2_ (κ = 3.9). In fact, even though the SiO_2_/3C-SiC interface is similar, in both cases resulting in analogous amount of effective charge (N_eff_), this can cause a variation of the experimental V_FB_ value, moving it toward the ideal one, as expressed by the following equations:

(1) $$\Delta V_{\mathrm{FB}}=\frac{{\mathrm{qN}}_{\mathrm{eff}}}{C_{\mathrm{OX}}},$$

(2) $$C_{\mathrm{OX}}=\frac{\varepsilon_{0\;}\kappa }{t_{\mathrm{OX}}},$$

where N_eff_ is the effective trapped charge density, C_OX_ is the accumulation capacitance, q is the electron charge, ε_0_ is the vacuum dielectric constant, and t_OX_ is the oxide thickness.

According to Equations (1) and (2), for a constant N_eff_ and an insulating layer thickness, an increased dielectric constant results in a smaller flat band voltage shift ΔV_FB_. Furthermore, the lower negative V_FB_ shift of the Al_2_O_3_/SiO_2_/3C-SiC stack can be also explained by the shorter time for the thermally oxidation process needed to grow a 4.5 nm SiO_2_ IL than that needed to grow a 30 nm thick SiO_2_. In fact, a shorter oxidation time produces a lower amount of carbon clusters responsible of the negative V_FB_ shift [38]. From the accumulation capacitance, the dielectric constant κ of the insulating films was estimated to be ~8 both for T- and PE-Al_2_O_3_. As can be seen in Figure 2b, the C-V curves of the PE- and T-ALD Al_2_O_3_/SiO_2_/3C-SiC are characterised by a different electrical behavior. In fact, besides the negative flat band voltage shift occurring in both cases, it can be noticed that a bump was visible in the depletion region of the C-V curve of the PE-ALD sample. Plausibly, this bump was caused by the occurrence of charge trapping at deep interface states when increasing the bias [12]. On the other hand, the thermal Al_2_O_3_/SiO_2_/3C-SiC sample is characterised by a more pronounced stretch-out of the C-V curve. Evidently, the different nature of the oxidation process (plasma enhanced-PE, and thermal-T) used during the ALD growth of Al_2_O_3_ was responsible for the different electrical quality of the two interfaces.

**Figure 3:**
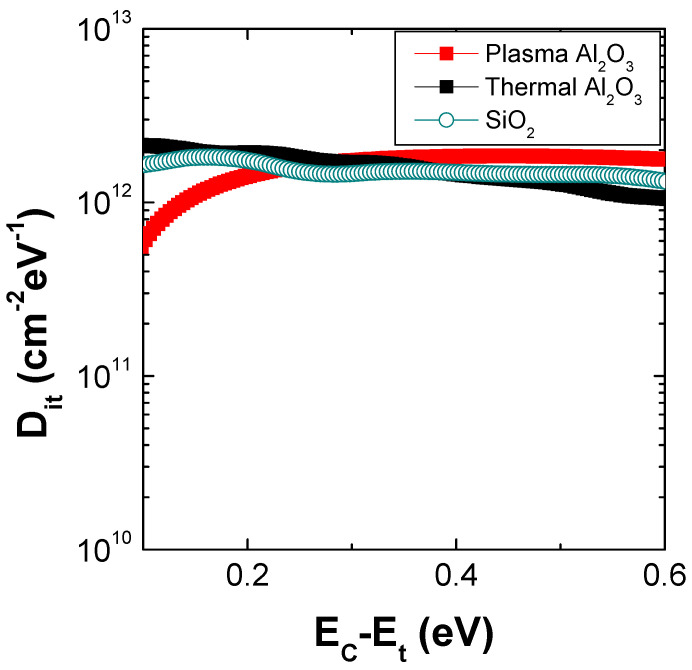
D_it_ distribution of T-Al_2_O_3_/SiO_2_(IL)/3C-SiC and PE-Al_2_O_3_/SiO_2_(IL)/3C-SiC MOS capacitors in comparison with the analogous SiO_2_/3C-SiC.

From the C-V curves, by applying the Terman’s method [39], the interface states (D_it_) distributions were calculated for both T-Al_2_O_3_/SiO_2_/3C-SiC and PE-Al_2_O_3_/SiO_2_/3C-SiC stacks, which are also reported in Figure 3 in comparison to that of the SiO_2_/3C-SiC system. The SiO_2_/3C-SiC and Al_2_O_3_/SiO_2_/3C-SiC samples exhibited a comparable D_it_ distribution in the order of 2 × 10^12^ cm^−2^ eV^−1^ [8]. On the other hand, the PE-Al_2_O_3_/SiO_2_/3C-SiC sample showed a lower D_it_ distribution close to the 3C-SiC conduction band edge in the order of 5 × 10^11^ cm^−2^ eV^−1^, which can be due to the beneficial effect of the O_2_-plasma on the defects amount at SiO_2_/SiC interface [40]. In fact, Kim et al. [30] demonstrated that for the SiO_2_/SiC-based devices, the use of a SiO_2_ growth process assisted by the highly reactive O_2_ plasma guarantees the formation of an interface characterised by a lower amount of defects and more stable SiO bonds. Analogously, in our case, the PE-approach used to deposit the Al_2_O_3_ could play a similar beneficial effect on the underlying SiO_2_/SiC interface.

**Figure 4:**
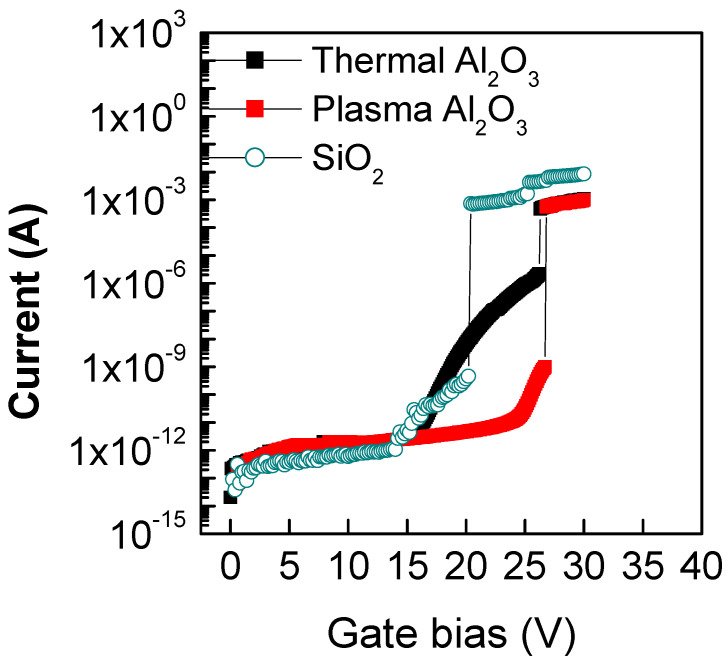
I-V measurements of T-Al_2_O_3_/SiO_2_/3C-SiC and PE-Al_2_O_3_/SiO_2_/3C-SiC MOS capacitors in comparison with the analogous SiO_2_/3C-SiC.

The current–voltage (I-V) curves acquired on the T-Al_2_O_3_/SiO_2_/3C-SiC and PE-Al_2_O_3_/SiO_2_/3C-SiC MOS capacitors are shown in Figure 4. As can be seen, in both systems, the electrical breakdown occurred at a gate bias of over 26 V. However, while the PE-Al_2_O_3_/SiO_2_/3C-SiC sample maintained a constant current value of 10^−12^ A up to the breakdown, the T-Al_2_O_3_/SiO_2_/3C-SiC sample exhibited a fast raise of the current starting from 15 V. The leakage current trend occurring across the Al_2_O_3_ layer deposited by thermal mode could be explained by a slightly lower mass density and a higher amount of -OH and/or -CH_3_ groups than that deposited by the plasma-enhanced mode due to the less efficacious oxidation process by the H_2_O-precursor [41,42,43]. Moreover, in comparison to the I-V curve typical of the 3C-SiC capacitor with a 40 nm thick SiO_2_ as a dielectric layer (also reported in Figure 4), which exhibited a breakdown voltage of about 20 V, both T-Al_2_O_3_/SiO_2_ and PE-Al_2_O_3_/SiO_2_ stacks were able to shift the breakdown phenomena toward higher voltages, over 26 V. The early breakdown of a thick thermal grown SiO_2_ on 3C-SiC has already been explained by F. Li et al. [44] as a consequence of the large amount of carbon left during the thermal oxidation process. However, in our case, the use of a short oxidation process to obtain only a thin IL probably resulted in a smaller amount of carbon defects to cause the early breakdown.

**Figure 5:**
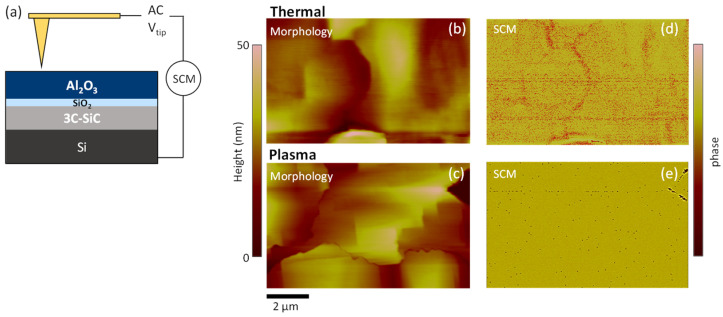
Schematic of the SCM experimental setup (**a**). AFM morphology of T-Al_2_O_3_/SiO_2_/3C-SiC (**b**) and PE-Al_2_O_3_/SiO_2_/3C-SiC (**c**). SCM maps of T-Al_2_O_3_/SiO_2_/3C-SiC (**d**) and PE-Al_2_O_3_/SiO_2_/3C-SiC (**e**).

The electrical behavior of both T- and PE-Al_2_O_3_/SiO_2_/3C-SiC stacks was studied at the nanoscale by SCM measurement. A schematic representation of the SCM experimental setup is illustrated in Figure 5a. During the surface scan with a diamond tip, an AC modulating bias at 100 kHz frequency and with amplitude ΔV = 2 V (below the conduction regime through the insulator) was applied to the sample, and the capacitance variation ΔC in response to this modulation was recorded with the SCM sensor. Figure 5b,c show the AFM morphology of the T- and PE-Al_2_O_3_/SiO_2_/3C-SiC stacks on the portion of the samples where the SCM maps were acquired. The highly irregular morphology is peculiar of the 3C-SiC material [18], which is characterised by terraces separated by anti-phase boundaries. The SCM maps of T- and PE-samples are reported in Figure 5d,e. The SCM signal is a result of the capacitance change (dC/dV) in the local metal-insulator-semiconductor capacitor, where the metal is the conductive AFM tip. Hence, the SCM response depends on the semiconductor characteristics (i.e., doping type and concentration) but also on the insulator properties (including thickness, interface state density, oxide traps, and permittivity) [45,46]. Considering that both the T- and PE-Al_2_O_3_ layers were deposited on the same 3C-SiC substrate and that they are characterised by an equivalent interface with SiO_2_ as IL, the different SCM maps (Figure 5d,e) obtained for the two cases can be correlated to the different insulator quality. In particular, the SCM map of the T-Al_2_O_3_/SiO_2_/3C-SiC stack, reported in Figure 5d, shows a non-uniform dC/dV signal distribution visible as the change in the color gradient from one spot to another. In contrast, the PE- Al_2_O_3_/SiO_2_/3C-SiC stack reported in Figure 5e exhibits a well-uniform SCM map, with only a small deviating region. The different SCM responses between T- and PE-systems could be due to the different structural quality of the Al_2_O_3_ layers deposited by the two approaches. In fact, the different Al_2_O_3_ quality, in terms of mass density and/or -OH/-CH_3_ contaminations, which can arise by using the different oxidation processes (T- or PE-), determines its charge trapping behavior and permittivity and, ultimately, the SCM signal. Similar results have been previously reported for the growth of Al_2_O_3_ thin layers on AlGaN/GaN heterostructures by the two different T-ALD and PE-ALD approaches, where the evolution of the insulating behavior investigated at the nanoscale upon increasing film thickness clearly indicated a different nucleation mechanism [16]. Hence, the present investigation at the nano-scale also confirms the better electrical performance of the PE-Al_2_O_3_ layer already observed by the electrical measurements acquired on the macroscopic capacitors.

## 4. Conclusions

The insulating properties of the Al_2_O_3_ layers deposited on 3C-SiC both by the thermal- and plasma-enhanced ALD approaches were investigated. Our results demonstrated that:

- A thin (5 nm) SiO_2_ IL between the Al_2_O_3_ and the 3C-SiC is useful to ensure the quality of ALD growth and to maximize the insulator/semiconductor band offset;
- The Al_2_O_3_ is a valid alternative to the conventional thermally grown single SiO_2_ as gate insulator for 3C-SiC MOS-based devices. In fact, the Al_2_O_3_ layers showed a high permittivity (~8), which produced a significant reduction in the negative flat band voltage shift that is usually observed with SiO_2_;
- A different electrical behavior was found between thermal- and plasma-enhanced Al_2_O_3_ both by investigations on macroscopic MOS capacitors and at the nanoscale using SCM analysis. In fact, although both systems ensure an electrical breakdown over 26 V, the T-Al_2_O_3_/SiO_2_/3C-SiC stack exhibits early leakage phenomena already from 15 V. Moreover, the T-Al_2_O_3_/SiO_2_/3C-SiC is characterised by a non-uniform SCM map compared to the PE-Al_2_O_3_/SiO_2_/3C-SiC. This difference can be correlated to a different Al_2_O_3_ quality obtained through the two different oxidation processes (T- or PE-), resulting in an inhomogeneous charge trapping behavior and permittivity.

These results can be important for the fabrication of 3C-SiC MOSFETs with a positive turn-on voltage with improved channel conduction properties.

## Data Availability

Data is contained within the article.

## Abbreviations

ALD	Atomic Layer Deposition
T-ALD	Thermal-ALD
PE-ALD	Plasma Enanched-ALD
NIOTs	Near Interface Oxide Traps
MOSFET	Metal Oxide Semiconductor Field Effect Transistor
MOS	Metal Oxide Semiconductor
IL	Interlayer
*High-κ*	High permittivity (κ) dielectrics
TEM	Transmission Electron Microscopy
AFM	Atomic Force Microscopy
SCM	Scanning Capacitance Microscopy
